# Short-Term Gastrointestinal Tolerance and Oxygenation Changes After a Locust Bean Gum-Containing Formula in Preterm Infants: A Retrospective Paired Cohort Study

**DOI:** 10.3390/nu18111834

**Published:** 2026-06-05

**Authors:** Murat Konak, Evrim Kılıçlı, Saime Sündüs Uygun, Havvanur Namal

**Affiliations:** Division of Neonatology, Department of Pediatrics, Selçuklu Medical Faculty, Selçuk University, Konya 42000, Türkiye; drevrimkilicli@gmail.com (E.K.); uygunsaime@hotmail.com (S.S.U.); dythemnur04@gmail.com (H.N.)

**Keywords:** locust bean gum, preterm infant, feeding tolerance, neonatal nutrition, oxygen saturation, anti-regurgitation formula, postmenstrual age

## Abstract

**Background/Objectives**: Gastrointestinal dysmotility, abdominal discomfort, and feeding-related respiratory instability are common in preterm infants. Although locust bean gum (LBG)-containing formulas are used for regurgitation, their short-term effects on gastrointestinal tolerance in neonatal intensive care settings are not well defined. We evaluated short-term changes in gastrointestinal tolerance and oxygenation after initiation of an LBG-containing formula and explored whether postmenstrual age (PMA) modified the response. **Methods**: This retrospective paired cohort study included 26 infants who received an LBG-containing anti-regurgitation formula, either alone or combined with human milk. Standardized ordinal scores (0–2) for stool consistency, straining, abdominal distension, gas passage, abdominal tenderness, mean oxygen saturation (SpO_2_) category, and desaturation frequency were recorded at baseline (Day 0), Day 3, and Day 7. Paired comparisons were performed using the Wilcoxon signed-rank test, and PMA-stratified differences were explored using Kruskal–Wallis analysis. **Results**: By Day 7, soft/normal stools were observed in 96.2% of infants (*p* = 0.00017), severe straining resolved in 87.5% (*p* = 1.6 × 10^−5^), abdominal distension improved in 96.2% (*p* = 2.4 × 10^−6^), and gas passage normalized in all infants (*p* = 0.00025). Mean SpO_2_ category improved significantly (*p* = 0.0023), and the proportion of infants with rare or no desaturation increased from 61.5% to 96.2%. Growth velocity remained clinically acceptable. Infants with PMA < 34 weeks showed the largest improvements across outcomes. **Conclusions**: In this retrospective paired cohort, initiation of an LBG-containing formula was associated with short-term improvement in gastrointestinal tolerance and oxygenation indices in preterm infants. Exploratory subgroup analyses suggested possible heterogeneity of response across postmenstrual age strata; these observations require confirmation in adequately powered prospective studies.

## 1. Introduction

Feeding intolerance, gastrointestinal dysmotility, and functional bowel discomfort are common problems in neonatal intensive care units, particularly among preterm infants. These disturbances may present with firm stools, straining, abdominal distension, irritability, and delayed feeding progression, thereby prolonging hospitalization and increasing clinical burden. Thickened formulas containing locust bean gum (LBG) are widely used to reduce gastroesophageal reflux and regurgitation in infancy by increasing feed viscosity [1,2,3,4]. However, in preterm infants, gastrointestinal symptoms often extend beyond reflux alone, and feeding-related discomfort may coexist with respiratory instability during routine care [5,6].

Several nutritional and pharmacologic approaches have been used to improve gastrointestinal tolerance in this population, but each has limitations. Prokinetic agents have been used in selected settings, although their safety and efficacy in preterm infants remain debated. By contrast, thickened formulas are already used in routine infant feeding practice, yet their effects have been evaluated mainly in the context of reflux reduction rather than broader bowel function. LBG, a galactomannan-based soluble fiber used in anti-regurgitation formulas, has properties that may be relevant to neonatal nutrition beyond viscosity modification [1,7]. Its water-binding capacity may contribute to softer stools and improved stool passage, while its physicochemical characteristics may also influence intraluminal handling of feeds [2,7]. At the same time, the effects of thickening agents are not uniform, and safety considerations may differ according to product composition and patient population [8,9].

Despite growing interest in LBG-containing formulas, the current literature has focused predominantly on regurgitation frequency, reflux control, or gastric emptying, with limited attention to clinically relevant bedside outcomes such as stool consistency, straining, abdominal distension, irritability, or gas passage [4,8]. Developmental maturity may also be important when interpreting feeding tolerance, because gastrointestinal motor function changes substantially across postmenstrual age (PMA) [10,11]. In preterm infants, therefore, both the potential benefits and the clinical response to thickened feeding strategies may differ according to maturational stage.

Against this background, we conducted a retrospective paired cohort study to evaluate short-term gastrointestinal tolerance and oxygenation changes after initiation of an LBG-containing anti-regurgitation formula in preterm infants. Using standardized clinical scores and paired within-infant comparisons, we also explored whether PMA modified the magnitude of response. We found that LBG formula initiation was associated with short-term improvement in several gastrointestinal tolerance parameters and oxygenation indices, with the largest changes observed in infants with lower PMA.

## 2. Materials and Methods

### 2.1. Study Design and Setting

This retrospective paired cohort study was conducted in a tertiary-level neonatal intensive care unit between October 2024 and November 2025. Clinical evaluations were performed at three predefined time points: baseline (Day 0), Day 3, and Day 7 after initiation of a locust bean gum (LBG)-containing formula. The study was designed to assess short-term within-infant changes in gastrointestinal tolerance and oxygenation parameters following introduction of the formula.

### 2.2. Formula Exposure

Infants received a commercially available anti-regurgitation formula containing locust bean gum (LBG; E410) at 0.35 g/100 mL (Aptamil AR^®^, Nutricia, Zoetermeer, The Netherlands). The formula was introduced either as the sole enteral feed or as a partial mixture with human milk, according to clinical judgment and feeding tolerance. Of the 26 infants included in the analysis, 19 (73.1%) received the LBG-containing formula as their exclusive enteral feed throughout the 7-day observation period, whereas 7 (26.9%) received it in combination with human milk at some point during follow-up. Among the latter, 3 infants received mixed feeding from Day 0 onward, whereas 4 transitioned to mixed feeding by Day 3 or Day 7. Before initiation of the LBG-containing formula, infants had been receiving standard preterm formula, fortified human milk, or mixed feeds without LBG. No infant received another thickening agent during the study period, and no xanthan gum-containing thickener was used.

### 2.3. Participants

Infants were eligible if they were hospitalized in the NICU during the study period, received a commercially available LBG-containing anti-regurgitation formula as part of clinical management [12,13], and had serial clinical assessments recorded between Day 0 and Day 7. Exclusion criteria were congenital gastrointestinal anomalies, necrotizing enterocolitis stage II or higher, postoperative ileus, requirement for escalation of respiratory support, and missing baseline or follow-up data for the primary outcome. One infant whose corrected postmenstrual age at the time of formula initiation substantially exceeded the preterm-relevant window was additionally excluded as a clinical outlier prior to analysis. A total of 26 infants met the eligibility criteria and were included in the final analysis.

### 2.4. Clinical Assessments

Gastrointestinal and respiratory outcomes were assessed using a standardized ordinal scoring system (0–2) integrated into local NICU practice. Gastrointestinal domains included stool frequency, stool consistency, gas passage, abdominal distension, abdominal tenderness, vomiting, and straining during feeding. Respiratory domains included desaturation frequency, apnea frequency, and mean oxygen saturation (SpO_2_) category. The ordinal scoring framework used in this study represents a locally adapted bedside assessment system integrated into routine NICU practice rather than a fully externally validated composite tool. The stool consistency component was informed by previously published infant stool assessment tools, particularly the Brussels Infant and Toddler Stool Scale [9,14]. The remaining domains reflected predefined routine clinical observation categories used in our NICU for daily charting. All assessments were performed by NICU nurses and neonatologists familiar with the scoring system through routine clinical use and staff orientation. However, formal interobserver variability testing was not conducted during the study period, and this is acknowledged as a methodological limitation.

The scoring categories were defined as follows: stool frequency, 0 = low, 1 = normal, 2 = optimal; stool consistency, 0 = pellet/firm, 1 = semi-formed, 2 = soft/normal; gas passage, 0 = absent, 1 = reduced, 2 = adequate; abdominal distension, 0 = marked, 1 = mild, 2 = none; abdominal tenderness, 0 = marked, 1 = mild, 2 = none; vomiting, 0 = frequent, 1 = occasional, 2 = none; desaturation frequency, 0 = frequent, 1 = occasional, 2 = rare/none; apnea frequency, 0 = frequent, 1 = occasional, 2 = rare/none; and mean SpO_2_ category, 0 = <90%, 1 = 90–94%, 2 = ≥95%. Straining during feeding was recorded using the same 0–2 ordinal framework, with higher scores indicating less straining.

### 2.5. Postmenstrual Age Stratification and Outcomes

Infants were stratified according to postmenstrual age (PMA) at formula initiation into three predefined groups: <34 weeks, 34–36 weeks, and ≥37 weeks. This stratification was selected a priori to explore whether maturational stage modified the clinical response to the nutritional intervention. The primary outcome was change in stool consistency score from Day 0 to Day 7. Secondary outcomes were changes in stool frequency, gas passage, abdominal distension, abdominal tenderness, vomiting, straining during feeding, desaturation frequency, apnea frequency, mean SpO_2_ category, and growth velocity. Body weight was recorded at Day 0, Day 3, and Day 7.

### 2.6. Statistical Analysis

Because most outcome variables were ordinal and the sample size was modest, non-parametric tests were used throughout. Paired Day 0 versus Day 7 comparisons were performed using the Wilcoxon signed-rank test. Differences across PMA strata were explored using the Kruskal–Wallis test with Dunn’s correction where appropriate. Analyses were performed using available paired observations for each variable. A two-sided *p* value < 0.05 was considered statistically significant.

### 2.7. Ethics Approval

The study was approved by the Local Ethics Committee of the Faculty of Medicine, Selçuk University (Decision No. 2025/318; approval date: 20 May 2025). The requirement for informed consent was waived because de-identified retrospective clinical data were used.

## 3. Results

### 3.1. Baseline Characteristics

A total of 26 infants were included in the analysis. Baseline demographic and clinical characteristics are summarized in Table 1. The cohort comprised 26 preterm infants with a balanced sex distribution (13 female and 13 male; 50.0% each). Each infant was followed for a total of 7 days, with paired clinical and respiratory assessments performed at Day 0, Day 3, and Day 7. Baseline demographic and clinical characteristics are summarized in Table 1. Birth weight ranged from 475 g to 2460 g (median 1187 g; IQR 995–1640), reflecting the inclusion of both very preterm and late-preterm infants, and median weight increased from 1952.5 g (IQR 1649–2476) on Day 0 to 2118 g (IQR 1863–2688) on Day 7. Infants initiated the locust bean gum (LBG)-containing formula across a broad postmenstrual age (PMA) range, and all completed the 7-day observation period. During follow-up, 19 infants (73.1%) received the LBG-containing formula as their exclusive enteral feed, whereas 7 (26.9%) received it in combination with human milk.

### 3.2. Changes in Gastrointestinal Tolerance and Oxygenation

**Figure 1:**
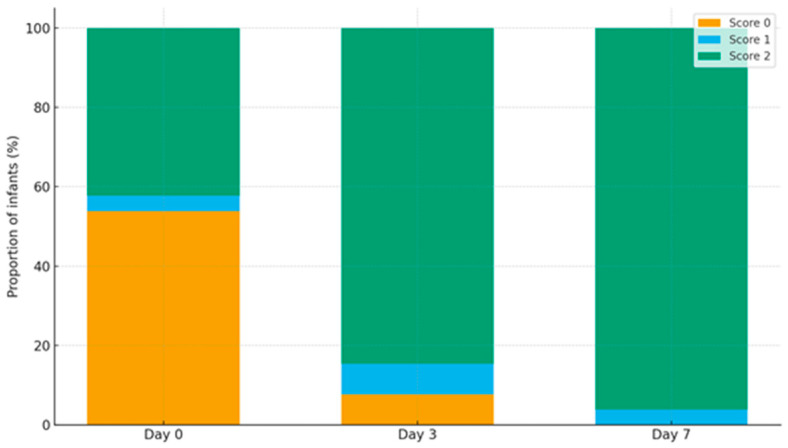
Stool consistency categories over time.

Short-term changes in gastrointestinal tolerance and oxygenation outcomes from Day 0 to Day 7 are summarized in Table 2. Stool consistency improved markedly over the study period. Pellet/firm stools present at baseline nearly disappeared by Day 7, and soft/normal stools were observed in 96.2% of infants (*p* = 0.00022). The distribution of stool consistency categories across study days is shown in Figure 1.

Gas passage also improved significantly, with all infants reaching the adequate category by Day 7 (*p* = 0.00025). Abdominal distension decreased markedly, and 96.2% of infants had no distension at Day 7 (*p* = 2.4 × 10^−6^). Abdominal tenderness improved in parallel (*p* = 6.7 × 10^−5^), and vomiting resolved completely by Day 7 (*p* = 0.0039). Stool frequency remained stable during follow-up (*p* = 0.059). This stability indicates that improvement in stool consistency was not accompanied by accelerated transit or osmotic effects, a pattern consistent with the water-binding rather than laxative properties of LBG.

**Figure 2:**
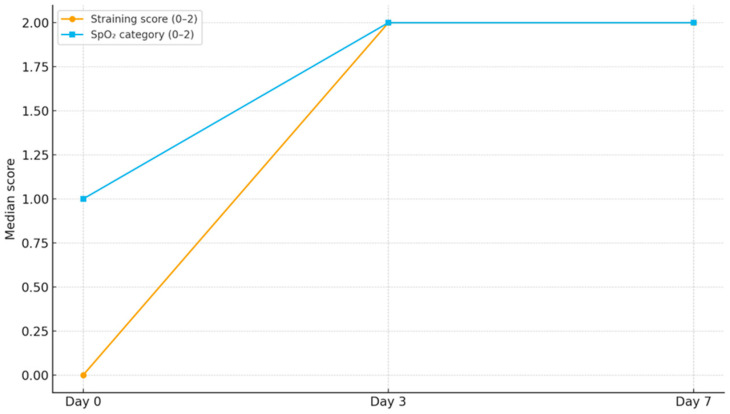
Median straining during feeding score and mean SpO_2_ category at Day 0, Day 3, and Day 7.

Straining during feeding improved substantially. Among infants with available paired data, severe straining observed at baseline was no longer present at Day 7, and most infants were in the best category by the end of follow-up (*p* = 1.6 × 10^−5^). Changes in straining during feeding and mean SpO_2_ category across the three assessment points are illustrated in Figure 2.

Respiratory indices also improved over the observation period. Mean SpO_2_ category increased significantly from Day 0 to Day 7 (*p* = 0.0023), and the proportion of infants with rare or no desaturation increased from 61.5% to 96.2% (*p* = 0.0282). In contrast, apnea score did not change significantly (*p* = 0.18). These findings are summarized in Table 2.

### 3.3. Exploratory PMA-Stratified Analyses

**Figure 3:**
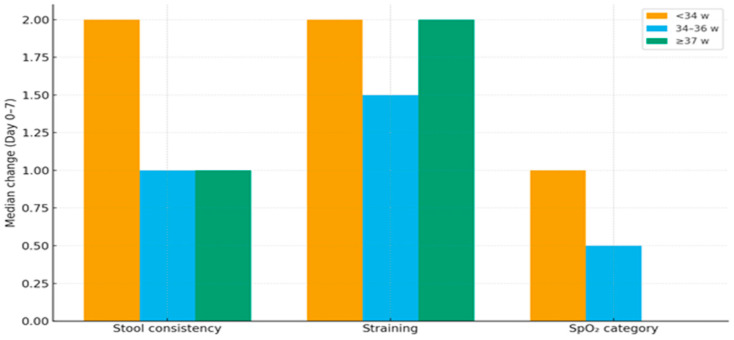
Exploratory PMA-stratified median changes in stool consistency, straining during feeding, and mean SpO_2_ category from Day 0 to Day 7. Higher delta values indicate greater observed improvement. Because subgroup sizes were small, these comparisons are descriptive and hypothesis-generating.

Infants were stratified into three PMA groups: <34 weeks (n = 5), 34–36 weeks (n = 8), and ≥37 weeks (n = 13). Numerically—but not statistically—greater median improvements in stool consistency, straining during feeding, and mean SpO_2_ category were observed in infants with lower PMA, particularly in the <34 weeks group. PMA-stratified changes in these clinical scores are shown in Figure 3. Given the small subgroup sizes and the absence of formal interaction testing, these analyses should be regarded as hypothesis-generating only and cannot be used to infer differential efficacy by maturational stage.

### 3.4. Growth Outcomes

Growth outcomes according to PMA group are presented in Table 3. Median Day 0–7 total weight gain was 180.0 g (IQR 155.0–230.0) in infants with PMA < 34 weeks, 212.5 g (IQR 112.5–292.5) in those with PMA 34–36 weeks, and 165.0 g (IQR 90.0–265.0) in those with PMA ≥ 37 weeks. Growth velocity remained within clinically acceptable ranges throughout the study period, and no infant discontinued the LBG-containing formula because of intolerance during follow-up. No adverse events were recorded during the observation period. Specifically, no episodes of suspected or confirmed necrotizing enterocolitis (Bell stage ≥ II), surgical abdominal events, or allergic-type reactions occurred. Bilious vomiting, frank blood in stool, and clinically significant abdominal distension requiring imaging did not arise in any infant.

## 4. Discussion

In this retrospective paired cohort of preterm infants, initiation of a locust bean gum (LBG)-containing formula was associated with rapid short-term improvement in several domains of gastrointestinal tolerance, including stool consistency, gas passage, abdominal distension, abdominal tenderness, vomiting, and straining during feeding. Mean SpO_2_ category and desaturation frequency also improved during the same observation period, whereas stool frequency and apnea score did not change significantly. Taken together, these findings suggest that LBG-containing formula may have broader short-term clinical associations than reflux control alone in selected NICU patients.

Previous studies of LBG-containing formulas have mainly focused on regurgitation, reflux reduction, gastric emptying, or general tolerance in infant feeding [1,3,4,8,15,16,17,18]. In contrast, data specifically addressing bedside gastrointestinal tolerance parameters in preterm infants remain limited. Our findings extend this literature by showing improvement in clinically relevant daily care variables, particularly stool consistency and straining during feeding, over a short observation period. Notably, stool frequency remained stable across the observation period, suggesting that the improvement in stool consistency reflected physiological softening through the water-binding properties of locust bean gum rather than an osmotic or laxative effect—a pattern compatible with favorable feeding tolerance in this vulnerable population. This interpretation is biologically plausible because LBG is a viscosity-modifying, water-binding fiber used in anti-regurgitation formulas [1,2,7,13,19,20]. However, our design does not allow mechanistic conclusions beyond these observed clinical associations.

The parallel improvement in oxygenation indices during the same observation period is intriguing but must be interpreted with substantial caution. Preterm infants commonly exhibit progressive improvement in respiratory stability over time due to maturation of central respiratory control, decreasing frequency of apnea of prematurity, and concurrent clinical management—including caffeine therapy, weaning of respiratory support, and optimization of positioning. Furthermore, the bidirectional relationship between gastroesophageal reflux, feeding, and apnea in early infancy is complex and not easily separated from maturational factors [21]. The present uncontrolled design cannot disentangle these maturational and management-related influences from any potential contribution of the feeding intervention. The oxygenation findings should therefore be regarded as a temporal clinical observation rather than as evidence of a formula-mediated respiratory benefit, and no causal gastrointestinal–respiratory mechanism can be inferred from the present data.

The exploratory PMA-stratified analyses also deserve cautious interpretation. Infants with lower PMA, particularly those <34 weeks, showed numerically—but not statistically—greater median changes in stool consistency, straining during feeding, and mean SpO_2_ category. This pattern is compatible with the general concept that developmental maturity influences gastrointestinal function and feeding tolerance [10,11,22,23]. Nevertheless, the subgroup sizes were small (n = 5, 8, and 13 in the three strata), and formal interaction testing was not performed. Given these limitations, the present data are insufficient to conclude that lower PMA independently predicts a stronger response to LBG-containing formula. These findings should therefore be regarded as preliminary signal generation only and cannot be used to infer differential efficacy by maturational stage.

This study has several strengths. It used a paired within-infant design, predefined assessment time points, and multidimensional clinical outcomes that reflect everyday gastrointestinal tolerance in neonatal practice. At the same time, important limitations must be acknowledged. A central limitation of this study is the absence of a contemporaneous control group. Preterm infants undergo rapid maturation of gastrointestinal motility, sucking coordination, and respiratory control during the postmenstrual age window covered by this cohort [10,11]. Consequently, part—or all—of the observed improvement in gastrointestinal tolerance and oxygenation indices may reflect natural maturational trajectories rather than a specific effect of the LBG-containing formula. The present findings should therefore be interpreted as hypothesis-generating clinical associations rather than as evidence of causality. Second, feeding exposure was heterogeneous: although the majority of infants (19 of 26; 73.1%) received the LBG-containing anti-regurgitation formula as their exclusive enteral feed throughout the 7-day observation period, a meaningful proportion (7 of 26; 26.9%) received the formula in combination with human milk at some point during follow-up. Human milk contains bioactive components—including human milk oligosaccharides, lactoferrin, and bile-salt-stimulated lipase—that independently support gastrointestinal motility, microbiota development, and mucosal homeostasis. Co-administration with human milk may therefore have either enhanced or attenuated the observed clinical response, and the independent effect of LBG exposure cannot be isolated within the present retrospective design. Formal subgroup comparison between exclusive-formula and mixed-feed strata was not undertaken because of small numbers in each cell. Third, outcomes were based on a locally adapted ordinal bedside scoring system rather than objective physiologic or motility measurements. Although the stool consistency component was informed by published infant stool assessment tools, the overall scoring framework was not a fully externally validated composite instrument. In addition, formal interobserver variability testing was not performed during the study period. Fourth, the sample size was modest and PMA subgroups were small, limiting statistical power and precluding formal interaction testing. Fifth, long-term outcomes including growth trajectory, neurodevelopment, and feeding behavior beyond Day 7 were not assessed. Finally, the study did not include dedicated safety surveillance for rare adverse gastrointestinal events. Although no adverse events were observed in the present cohort, continued pharmacovigilance for rare complications—such as those previously reported in association with xanthan-gum-containing thickeners [24]—is warranted in future prospective studies. These issues limit causal inference and generalizability.

Prospective controlled studies are needed to determine whether these associations persist after adjustment for maturation and concurrent feeding practices, and to clarify whether any respiratory benefit is reproducible in larger neonatal cohorts.

## 5. Conclusions

In this retrospective paired cohort, initiation of a locust bean gum (LBG)-containing formula was associated with short-term improvement in several clinically relevant gastrointestinal tolerance parameters in preterm infants, including stool consistency, gas passage, abdominal distension, abdominal tenderness, vomiting, and straining during feeding. Improvement in mean SpO_2_ category and desaturation frequency was also observed during the same period, although these findings should be interpreted cautiously given the uncontrolled design. Infants with lower postmenstrual age showed numerically—but not statistically—greater improvement across selected outcomes, but these exploratory subgroup findings require confirmation in adequately powered prospective trials. Overall, these results support further prospective controlled studies to clarify the nutritional and clinical effects of LBG-containing formulas in preterm infants.

## Figures and Tables

**Table 1:** Baseline demographic and characteristics of the study cohort (n = 26).

Characteristic	Value
Gestational age, weeks	29.3 ± 3.1 (median 28.2; range 24.1–36.2)
Postmenstrual age at AR initiation, weeks	36.6 ± 3.0 (median 36.95; range 30.2–42.5)
Birth weight, g	1293 ± 489 (median 1187.5; range 475–2460)
Sex, n (%)	
Female	13 (50.0%)
Male	13 (50.0%)
Feeding exposure during the 7-day observation period, n (%)	
Exclusive LBG-containing formula throughout follow-up	19 (73.1%)
LBG-containing formula + human milk at any time during follow-up	7 (26.9%)
Mixed from Day 0 onward	3 (11.5%)
Transitioned to mixed feeding by Day 3 or Day 7	4 (15.4%)

Values are presented as median (interquartile range, IQR) unless otherwise indicated.

**Table 2:** Changes in gastrointestinal tolerance and oxygenation outcomes from Day 0 to Day 7.

Outcome	Day 0 Median (IQR)	Day 3 Median (IQR)	Day 7 Median (IQR)	*p*-Value (Day 0 vs. Day 7)
Stool frequency score (0–2)	2.0 (2.0–2.0)	2.0 (2.0–2.0)	2.0 (2.0–2.0)	0.059
Stool consistency score (0–2)	0.0 (0.0–2.0)	2.0 (2.0–2.0)	2.0 (2.0–2.0)	0.00022
Gas passage score (0–2)	1.0 (0.0–2.0)	2.0 (2.0–2.0)	2.0 (2.0–2.0)	0.00025
Abdominal distension score (0–2)	0.0 (0.0–0.0)	2.0 (1.0–2.0)	2.0 (2.0–2.0)	2.4 × 10^−6^
Abdominal tenderness score (0–2)	0.5 (0.0–1.8)	2.0 (2.0–2.0)	2.0 (2.0–2.0)	6.7 × 10^−5^
Vomiting score (0–2)	2.0 (1.0–2.0)	2.0 (2.0–2.0)	2.0 (2.0–2.0)	0.0039
Desaturation score (0–2)	2.0 (1.0–2.0)	2.0 (2.0–2.0)	2.0 (2.0–2.0)	0.0282
Straining during feeding score (0–2) *	0.0 (0.0–0.0)	2.0 (0.8–2.0)	2.0 (2.0–2.0)	1.6 × 10^−5^
Apnea score (0–2)	2.0 (2.0–2.0)	2.0 (2.0–2.0)	2.0 (2.0–2.0)	0.18
Mean SpO_2_ category score (0–2)	1.0 (1.0–1.8)	2.0 (1.0–2.0)	2.0 (1.0–2.0)	0.0023

* Available paired data for straining during feeding: n = 24. Wilcoxon signed- rank test was used for paired Day 0 versus Day 7 comparisons. Scores ranged from 0 (worst) to 2 (best) for all outcomes.

**Table 3:** Growth outcomes according to PMA group.

PMA Group	n	Day 0–3 Velocity (g/Day), Median (IQR)	Day 3–7 Velocity (g/Day), Median (IQR)	Day 0–7 Total Gain (g), Median (IQR)
<34 weeks	5	13.3 (8.3–20.0)	30.0 (28.8–35.0)	180.0 (155.0–230.0)
34–36 weeks	8	23.3 (20.0–33.3)	38.1 (17.5–42.8)	212.5 (112.5–292.5)
≥37 weeks	13	26.7 (16.7–51.7)	15.0 (5.0–30.0)	165.0 (90.0–265.0)

Velocity values are expressed as grams per day (g/day).

## Data Availability

The data presented in this study are available on reasonable request from the corresponding author. The data are not publicly available due to privacy and ethical restrictions, as the study includes retrospective clinical data from preterm infants. A de-identified minimal dataset supporting the main findings can be provided for editorial review and, where appropriate, shared upon reasonable request.

## Abbreviations

The following abbreviations are used in this manuscript:

LBG	locust bean gum
NICU	neonatal intensive care unit
PMA	postmenstrual age
SpO_2_	oxygen saturation

## References

[1] Salvatore S., Klymenko V., Karpushenko Y., Durczak-Hilleman M., Loboda A., Petrashenko V., Olechowski W., Lista G., Meneghin F., Amodio S. (2024). Tolerance and safety of an anti-regurgitation formula containing locust bean gum, pre-, and postbiotics: A multi-country multi-center prospective randomized controlled study in infants with regurgitation. Nutrients.

[2] Ikram A., Khalid W., Wajeeha Zafar K., Ali A., Afzal M.F., Aziz A., Faiz ul Rasool I., Al-Farga A., Aqlan F., Koraqi H. (2023). Nutritional, biochemical, and clinical applications of carob: A review. Food Sci. Nutr..

[3] Salvatore S., Savino F., Singendonk M., Tabbers M., Benninga M.A., Staiano A., Vandenplas Y. (2018). Thickened infant formula: What to know. Nutrition.

[4] Miyazawa R., Tomomasa T., Kaneko H., Morikawa A. (2006). Effect of formula thickened with locust bean gum on gastric emptying in infants. J. Paediatr. Child Health.

[5] Arslan S.S. (2021). Yenidoğan Döneminde Yutma Bozukluklarının Değerlendirilmesi ve Tedavisi. Akdeniz Tıp Derg..

[6] Corvaglia L., Mariani E., Aceti A., Galletti S., Faldella G. (2013). Extensively hydrolyzed protein formula reduces acid gastro-esophageal reflux in symptomatic preterm infants. Early Hum. Dev..

[7] Meunier L., Garthoff J.A., Schaafsma A., Krul L., Schrijver J., van Goudoever J.B., Speijers G., Vandenplas Y. (2014). Locust bean gum safety in neonates and young infants: An integrated review of the toxicological database and clinical evidence. Regul. Toxicol. Pharmacol..

[8] Bellaiche M., Tounian P., Oozeer R., Rocher E., Vandenplas Y. (2023). Digestive tolerance and safety of an anti-regurgitation formula containing locust bean gum, prebiotics and postbiotics: A real-world study. Pediatr. Gastroenterol. Hepatol. Nutr..

[9] Vandenplas Y., Szajewska H., Benninga M., Di Lorenzo C., Dupont C., Faure C., Miqdadi M., Osatakul S., Ribes-Konickx C., Saps M. (2017). Development of the Brussels Infant and Toddler Stool Scale (‘BITSS’): Protocol of the study. BMJ Open.

[10] Indrio F., Riezzo G., Raimondi F., Cavallo L., Francavilla R. (2009). Regurgitation in healthy and non healthy infants. Ital. J. Pediatr..

[11] Berseth C. (1989). Gestational evolution of small intestine motility in preterm and term infants. J. Pediatr..

[12] Younes M., Aquilina G., Castle L., Degen G., Engel K.H., Fowler P.J., Frutos Fernandez M.J., Fürst P., Gürtler R., EFSA Panel on Food Additives and Flavourings (FAF) (2023). Re-evaluation of locust bean gum (E 410) as a food additive in foods for infants below 16 weeks of age and follow-up of its re-evaluation as a food additive for uses in foods for all population groups. EFSA J..

[13] Mortensen A., Aguilar F., Crebelli R., Di Domenico A., Frutos M.J., Galtier P., Gott D., Gundert-Remy U., Lambré C., EFSA Panel on Food Additives Nutrient Sources added to Food (ANS) (2017). Re-evaluation of locust bean gum (E 410) as a food additive. EFSA J..

[14] Huysentruyt K., Koppen I., Benninga M., Cattaert T., Cheng J., De Geyter C., Faure C., Gottrand F., Hegar B., Hojsak I. (2019). The Brussels Infant and Toddler Stool Scale: A study on interobserver reliability. J. Pediatr. Gastroenterol. Nutr..

[15] Tounian P., Meunier L., Speijers G., Oozeer R., Vandenplas Y. (2020). Effectiveness and tolerance of a locust bean gum-thickened formula: A real-life study. Pediatr. Gastroenterol. Hepatol. Nutr..

[16] Georgieva M., Manios Y., Rasheva N., Pancheva R., Dimitrova E., Schaafsma A. (2016). Effects of carob-bean gum thickened formulas on infants’ reflux and tolerance indices. World J. Clin. Pediatr..

[17] Vandenplas Y. (2009). Thickened infant formula does what it has to do: Decrease regurgitation. Pediatrics.

[18] Vivatvakin B., Buachum V. (2003). Effect of carob bean on gastric emptying time in Thai infants. Asia Pac. J. Clin. Nutr..

[19] Baert K., Ombecq M., Van Winckel M., Henry S., Tommelein E., Vanhoorne V. (2024). The viscosity-enhancing effect of carob bean gum and sodium carboxymethylcellulose when added to infant formula. Food Sci. Nutr..

[20] Koo J.K., Narvasa A., Bode L., Kim J.H. (2019). Through thick and thin: The in vitro effects of thickeners on infant feed viscosity. J. Pediatr. Gastroenterol. Nutr..

[21] Slocum C., Hibbs A.M., Martin R.J., Orenstein S.R. (2007). Infant apnea and gastroesophageal reflux: A critical review and framework for further investigation. Curr. Gastroenterol. Rep..

[22] Mihatsch W.A., Franz A.R., Högel J., Pohlandt F. (2002). Hydrolyzed protein accelerates feeding advancement invery low birth weight infants. Pediatrics.

[23] Mihatsch W., Högel J., Pohlandt F. (2001). Hydrolysed protein accelerates the gastrointestinal transport of formula in preterm infants. Acta Paediatr..

[24] Beal J., Silverman B., Bellant J., Young T.E., Klontz K. (2012). Late onset necrotizing enterocolitis in infants following use of a xanthan gum-containing thickening agent. J. Pediatr..

